# Small Drusen and Age-Related Macular Degeneration: The Beaver Dam Eye Study

**DOI:** 10.3390/jcm4030425

**Published:** 2015-03-09

**Authors:** Ronald Klein, Chelsea E. Myers, Kristine E. Lee, Ronald E. Gangnon, Theru A. Sivakumaran, Sudha K. Iyengar, Barbara E. K. Klein

**Affiliations:** 1 Department of Ophthalmology and Visual Sciences, University of Wisconsin School of Medicine and Public Health, Madison, WI 53726, USA; E-Mails: myers@epi.ophth.wisc.edu (C.E.M.); klee@epi.ophth.wisc.edu (K.E.L.); kleinb@epi.ophth.wisc.edu (B.E.K.K.); 2 Department of Biostatistics and Medical Informatics and Department of Population Health Sciences, University of Wisconsin School of Medicine and Public Health, Madison, WI 53726, USA; E-Mail: ronald@biostat.wisc.edu; 3 Department of Epidemiology and Biostatistics, Case Western Reserve University, Cleveland, OH 44106, USA; E-Mails: sth248@uky.edu (T.A.S.); ski@case.edu (S.K.I.); 4 Department of Pathology and Laboratory Medicine, University of Kentucky College of Medicine, Lexington, KY 40536, USA

**Keywords:** age-related macular degeneration, epidemiology, genetic risk, incidence, retinal drusen, risk factors, size and area

## Abstract

We tested the hypothesis that large areas of small hard drusen (diameter <63 µm) and intermediate drusen (diameter 63–124 µm) are associated with the incidence of age-related macular degeneration (AMD). Eyes of 3344 older adults with at least two consecutive visits spaced five years apart over a 20-year period were included. A 6-level severity scale, including no drusen, four levels of increasing area (from minimal (<2596 µm²) to large (>9086 µm²)) of only small hard drusen, and intermediate drusen, was used. The five-year incidence of AMD was 3% in eyes at the start of the interval with no, minimal, small, and moderate areas of only small drusen and 5% and 25% for eyes with large area of only small drusen and intermediate drusen, respectively. Compared to eyes with a moderate area of small drusen, the odds ratio (OR) of developing AMD in eyes with a large area of only small drusen was 1.8 (*p* < 0.001). Compared to eyes with large area of only small drusen, eyes with intermediate drusen had an OR of 5.5 (*p* < 0.001) of developing AMD. Our results are consistent with our hypothesis that large areas of only small drusen are associated with the incidence of AMD.

## 1. Introduction

Age-related macular degeneration (AMD) is a complex chronic disease affecting multiple layers of the retina. Histologically, its earliest subclinical stages have been defined by the presence of age-related thickening of Bruch membrane with calcifications and basal laminar deposits prior to the appearance of hard and soft drusen in the macula as detected by ophthalmoscopy [1]. Clinically, its earliest stages are often defined by the presence of large soft drusen (≥125 µm in diameter) [2,3]. Progression of AMD involves the appearance of increasing numbers of large soft distinct and indistinct drusen that may become confluent, as well as the development of pigmentary abnormalities (depigmentation and increased retinal pigment) of the retinal pigment epithelium (RPE) in the macula. Progression rates vary, with some eyes remaining in early stages of the disease over long periods of time while others progress more rapidly to the late stages of the disease, geographic atrophy and neovascular AMD. Reduction in choroidal perfusion and retinal tissue oxygenation levels, increased systemic inflammation, presence of atherosclerosis, and elastic tissue degeneration have been hypothesized to be involved in the pathogenesis of AMD [3,4]. Genes associated with the alternative complement system, lipid metabolism, atherosclerosis, and other pathways have been found to be associated with the development of late AMD [5,6].

Labeling an eye as having early AMD has been based on empirical evidence from epidemiological studies (e.g., the Beaver Dam Eye Study, the Rotterdam Study, the Blue Mountains Eye Study), clinical trials, such as the Age-Related Eye Disease Study (AREDS) and observations of clinical cohorts [2,3,7,8,9,10,11,12]. In those studies, the presence of large drusen (≥125 µm in diameter) in one or both eyes increased the risk of developing signs of late AMD [2,3,11]. In the AREDS, the presence of intermediate drusen (between 63 and 124 µm in diameter) in both eyes was also associated with a small but significant increase in risk of developing late AMD [2,11]. Presence of a few small hard drusen is common and is not thought to be an early stage of AMD [13,14]. Eyes with a larger area of only small hard drusen (>9086 µm^2^) compared with eyes with a smaller drusen area (<2596 µm²) involving the macula have been found to be associated with increased age-adjusted 15-year cumulative incidence of soft indistinct drusen (16.3% *vs.* 4.7%) and pigmentary abnormalities (10.6% *vs.* 2.7%) [7]. These findings suggested that a larger area of small hard drusen in the absence of larger soft drusen or other retinal AMD lesions in an eye might be an even earlier clinical stage of AMD than that which is currently defined by the presence of larger drusen.

We hypothesized that larger areas of only small hard drusen and the presence of intermediate drusen were related to the incidence of AMD. We also hypothesized that age, sex, and two specific single-nucleotide polymorphisms (SNPs), one in the complement factor H region (CFH Y402H rs1061170) on chromosome 1 and the other in the age-related maculopathy susceptibility 2 region (ARMS2 A69S rs10490924) on chromosome 10, were related to the incidence of increasing areas of small hard drusen and to intermediate drusen. Longitudinal data over a 20-year period from the Beaver Dam Eye Study were used in the analyses.

## 2. Experimental Section

### 2.1. Study Population

Identification and descriptions of the population in the Beaver Dam Eye Study have appeared in previous reports [15,16,17,18,19,20]. In brief, a private census identified 5924 persons living in the city and township of Beaver Dam, Wisconsin aged 43–86 years, of which 4926 participated in the baseline examination in 1988–1990 and in up to four follow-up examinations in 1993–1995, 1998–2000, 2003–2005 and 2008–2010. Over 99% of the population was white. All subjects gave their informed consent for inclusion before they participated in the study. The investigations were carried out following the rules of the Declaration of Helsinki, and the research protocol was approved by the institutional review board at the University of Wisconsin-Madison. Comparisons between participants and nonparticipants at each examination have appeared elsewhere [15,16,17,18,19,20]. In general, those who participated in a follow-up examination over the 20-year period of the study were more likely to be younger than nonparticipants who were alive or those who died before follow-up and, with adjustment for age, were less likely to have early AMD as defined as having severity level 20 or greater on the 5-step Three Continent Consortium AMD severity scale in at least 1 eye [9].

### 2.2. Procedures

The baseline and follow-up examinations used similar procedures, which have been described in detail elsewhere [2,4,13,21,22,23,24,25,26,27]. Stereoscopic 30° color film fundus photographs centered on the disc (Diabetic Retinopathy Study standard field 1) and macula (Diabetic Retinopathy Study standard field 2) and a nonstereoscopic color fundus photograph temporal to but including the fovea of each eye were obtained. Details of the grading procedure have been described elsewhere [2,4,13,21,22,23,24,25,26,27].

### 2.3. Genetic Measurements

The genetic measurements have been described in detail elsewhere [28]. In brief, samples of DNA were extracted from buffy coat specimens collected at the baseline examination. The two SNPs most commonly associated with AMD, Y402H in CFH (rs1061170) and A69S in ARMS2 (rs10490924), were used in this study. For CFH, T was considered the wild allele and C the risk allele; for ARMS2, G was considered the wild allele and T the risk allele.

### 2.4. Definitions

Age was documented at each participant visit and was categorized as <60 years, 60–69 years, or ≥70 years. Body mass index was calculated by dividing a participant’s weight in kilograms by his or her height in meters squared. Current heavy drinking was defined as the consumption of 4 or more servings of alcoholic beverages daily at the time of the examination; a serving was defined as 12 fluid ounces of beer, 4 fluid ounces of wine, or 1.5 fluid ounces of liquor. Participants were considered physically active if they engaged in physical activity long enough to work up a sweat at least 3 times per week. Hypertension was defined as blood pressure ≥140/90 mmHg or use of anti-hypertensive medication. Aspirin, multivitamin, and anti-hypertensive medication use were determined from self-report.

The severity status of drusen in an eye was defined as follows.

- **Level 1 (No drusen):** No signs of any drusen or of early or late AMD.
- **Level 2 (Minimal area of small hard distinct drusen):** Presence of small hard distinct drusen <63 µm in diameter as the largest size drusen involving a circular area of the macula of up to 2596 µm² and no other signs of AMD.
- **Level 3 (Small area of small hard distinct drusen):** Presence of small hard distinct drusen <63 µm in diameter as the largest size drusen involving a circular area of the macula between 2597 µm² and 5192 µm² and no other signs of AMD.
- **Level 4 (Moderate area of small hard distinct drusen):** Presence of small hard distinct drusen <63 µm in diameter as the largest size drusen involving a circular area of the macula between 5193 µm² and 9086 µm² and no other signs of AMD.
- **Level 5 (Large area of small hard distinct drusen):** Presence of small hard distinct drusen <63 µm in diameter as the largest size drusen involving a circular area of the macula greater than 9086 µm² and no other signs of AMD.
- **Level 6 (Intermediate drusen):** Presence of one or more drusen 63 to 124 µm in diameter as the largest size drusen and no other signs of AMD.

Early AMD was defined as the presence of small to intermediate drusen, regardless of area of involvement, with any pigmentary abnormalities (defined as depigmentation and increased retinal pigment) or the presence of large drusen (≥125 µm in diameter). Late AMD was defined by the presence of geographic atrophy and signs of exudative AMD, such as serous detachment of the RPE and/or sensory retina.

### 2.5. Statistical Analysis

We examined the association of the drusen severity level in an eye and other risk factors measured at one examination with drusen severity level in that eye at the next examination, 5 years later. Pairs of visits were accumulated during the 20 years of the study and risk factors were updated for each 5-year period. Multiple contributions from an individual were accounted for by using generalized estimating equations (GEE).

An eye was considered at risk for incidence of a specific level on the drusen severity scale if drusen were gradable at 2 consecutive visits and had a less severe level of drusen at the earlier examination. The outcome of interest was the development of drusen at or above a given severity level (e.g., intermediate drusen or any AMD). For example, development of intermediate drusen (Level 6) was evaluated for all eyes with Level 1 through Level 5 at the start of an interval. If the severity level was Level 6 or any AMD at the follow-up visit, the eye was considered to have developed intermediate drusen.

Analyses of specific risk factors (age, sex, genotype) were stratified by the initial drusen severity level. Models were adjusted for age and sex. SAS version 9.2 (SAS Institute Inc., Cary, NC, USA) was used for all analyses.

## 3. Results

### 3.1. Participant Characteristics

There were 3344 individuals who contributed AMD data for at least one pair of consecutive examinations: 3198 with baseline and five-year follow-up data, 2367 participants with five-year and 10-year follow-up data, 1865 participants with 10-year and 15-year follow-up data, and 1468 participants with 15-year and 20-year follow-up data. For each interval, 75%–80% of individuals had data available for both eyes, resulting in 15821 total person-eye-visit intervals. Table 1 shows the characteristics of this group. The percentages of individuals at the start of a five-year follow-up interval with Level 1, Level 2, Level 3, Level 4, Level 5, and Level 6 were 14%, 26%, 16%, 15%, 14% and 16%, respectively (Table 1). The genotype distributions for CFH Y402H were 41%, 46% and 13% for T/T, T/C, and C/C, respectively; the genotype distributions for ARMS2 A69S were 61%, 35%, and 4% for G/G, G/T, and T/T, respectively.

### 3.2. Incidence of Varying Areas of Small Hard Drusen, Intermediate Drusen, and Any AMD

**Figure 1:**
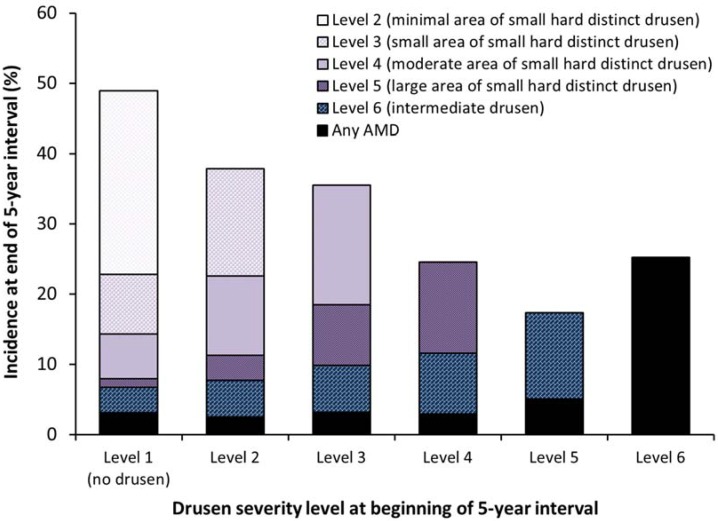
The relationship of no drusen, 4 areas of increasing involvement of the macula by small hard drusen and intermediate drusen to the 5-year incidence of more severe levels of drusen and any AMD in the Beaver Dam Eye Study, 1988–1990 to 2008–2010.

Figure 1 shows the distribution of drusen severity levels at the follow-up examination for each level at the start of the interval and Table 2 shows the results of incidence analyses for each drusen severity level. As severity level at the beginning of an interval increased, so did the development of the more severe drusen levels (Figure 1). For example, 8% of eyes with Level 1 (*n* = 2206) at the start of a five-year follow-up interval developed Level 5 or worse compared with 25% of eyes starting at Level 4 (*n* = 2370). 49% of eyes with Level 1 (*n* = 2206) developed Level 2 or worse by the end of a five-year period, and 36% of eyes with Level 3 (*n* = 2515) developed Level 4 or worse.

Five-year incidence of Level 6 or worse was 17%, 12%, 10%, 8%, and 7% for individuals with Level 5, Level 4, Level 3, Level 2, and Level 1 at the start of the interval, respectively. Incidence of any AMD was higher in eyes with Level 6 at the start of the interval compared to eyes with Level 5 (25% *vs.* 5%; odds ratio [OR] = 5.5) and was also higher in eyes with Level 5 *vs.* Level 4 at the beginning of a five-year period (OR = 1.8).

**Table 1:** Characteristics of individuals included in analyses averaged over all examination phases in the Beaver Dam Eye Study, 1988–1990 to 2008–2010. *ARMS2*, age-related maculopathy susceptibility 2 rs10490924; *CFH*, complement factor H rs1061170; SD, standard deviation.

Characteristic		Person-Eye-Visits (*N*)	Mean (SD) or %
Age, years		15821	62.3 (9.5)
Sex	Female	9016	57.0
	Male	6805	43.0
Beginning drusen severity level	Level 1	2206	13.9
	Level 2	4055	25.6
	Level 3	2515	15.9
	Level 4	2370	15.0
	Level 5	2210	14.0
	Level 6	2465	15.6
*CFH* genotype	T/T	5942	40.6
	T/C	6771	46.3
	C/C	1919	13.1
*ARMS2* genotype	G/G	9172	60.9
	G/T	5323	35.3
	T/T	574	3.8
Hypertension present	No	7911	50.4
	Yes	7776	49.6
Body mass index, kg/m²		15625	29.6 (5.6)
Smoking history	Never	7312	46.2
	Past	6189	39.1
	Current	2312	14.6
Heavy drinking history	Never	13377	84.6
	Past	2154	13.6
	Current	276	1.7
Physical activity level	Active	4843	30.6
	Sedentary	10969	69.4
Using multivitamins	No	7720	48.8
	Yes	8101	51.2
Currently using aspirin	No	10946	69.3
	Yes	4854	30.7

### 3.3. Relationships of Age, Sex and Two AMD Candidate Genes to the Five-Year Incidences of Small Hard Drusen, Intermediate Drusen and AMD

**Figure 2:**
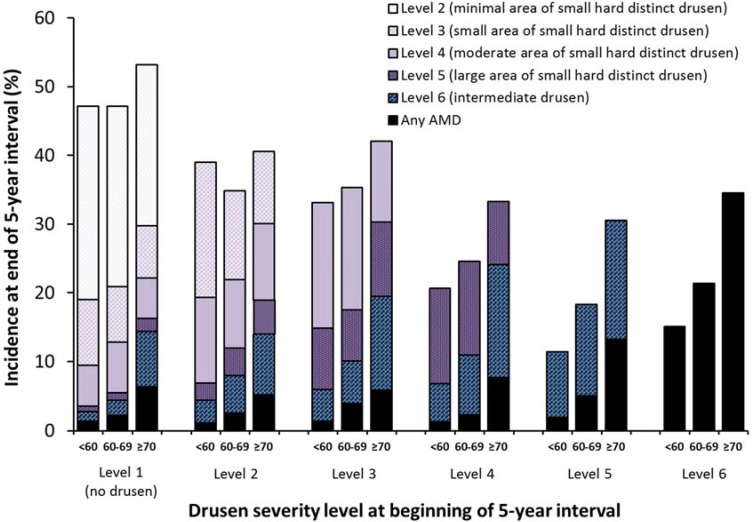
Five-year incidence of increasing area of small hard drusen, intermediate drusen, and any AMD by age group (<60, 60–69, and ≥70 years) and severity level at the beginning of the interval.

**Figure 3:**
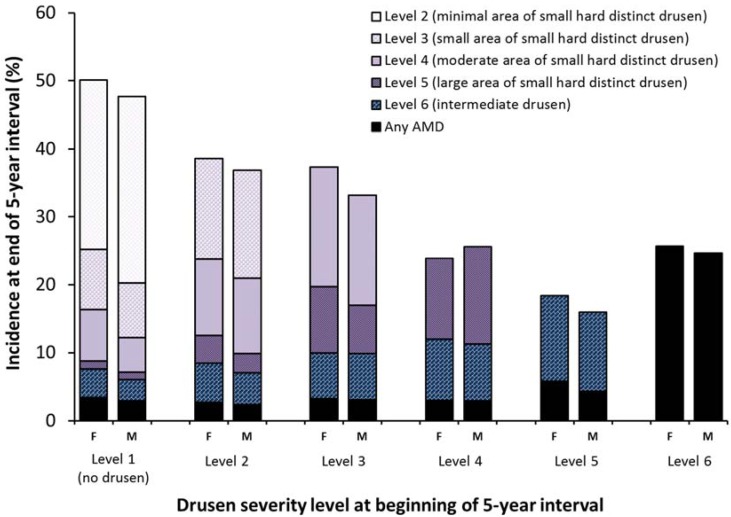
Five-year incidence of increasing area of small hard drusen, intermediate drusen, and any AMD by sex and severity level at the beginning of the interval. F = female; M = male.

**Figure 4:**
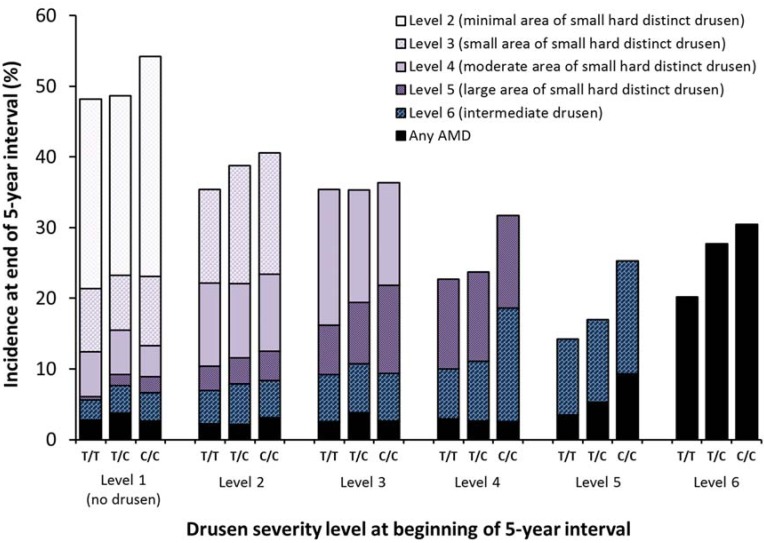
Five-year incidence of increasing area of small hard drusen, intermediate drusen, and any AMD by CFH Y402H (rs1061170) genotype and severity level at the beginning of the interval.

**Figure 5:**
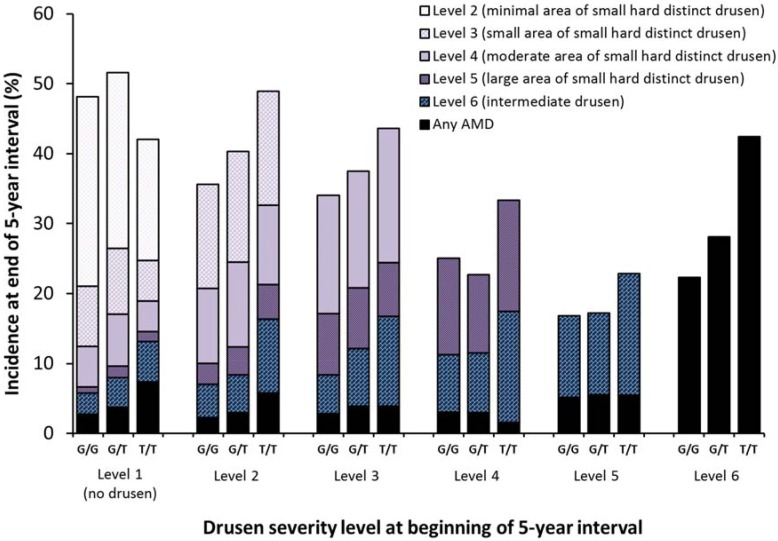
Five-year incidence of increasing area of small hard drusen, intermediate drusen, and any AMD by ARMS2 A69S (rs10490924) genotype and severity level at the beginning of the interval.

Figure 2, Figure 3, Figure 4, Figure 5 and Supplementary Tables S1–S6 show the relationships between drusen severity level and AMD status at follow-up by age, sex and genetic information, stratified by initial drusen level. Figure 2 shows the distribution of the follow-up drusen severity level and AMD status by age group. Odds ratios for these relationships are presented in Supplementary Tables S1–S6, with each possible outcome in a separate table. Older age was associated with higher five-year incidence of most outcomes and the associations were stronger in eyes with larger areas of small hard drusen and more severe lesions. Sex was not associated with the development of any drusen outcome (Figure 3).

Figure 4 and Figure 5 and Supplementary Tables S1–S6 show the relationships of the CFH and ARMS2 candidate genes stratified by drusen severity levels at the beginning of an interval to the five-year incidences for each drusen severity level and AMD outcome. While few individuals had two risk alleles, especially for ARMS2, the increasing incidence was still apparent and statistically significant for some relationships. For CFH Y402H genotype (Figure 3), the strongest associations for incidence of Level 6 (Supplementary Table S5) and incidence of any AMD (Supplementary Table S6) occurred when the initial severity was at least Level 4. Incidence of Level 6 was about 9%–10% regardless of the number of risk alleles for CFH Y402H (*p* = 0.35 for any differences after adjusting for age and sex) when initial drusen severity was Level 3 but ranged from 14% to 25% for 0 *vs.* 2 risk alleles when the initial severity was Level 5 (OR = 1.4 per risk allele, *p* < 0.001 after adjusting for age and sex). Where an association with CFH was present, the association was stronger in individuals with 2 risk alleles compared to individuals with only one risk allele.

**Table 2:** Incidence of specific level of drusen area/severity by initial severity level. *N* is the number of person-visit-eyes at risk. The *p* value is the Wald test for any differences between levels. AMD, age-related macular degeneration; CI, confidence interval; OR, odds ratio.

Incidence of	Beginning Level	*N* at Risk	% Incident	Comparison Level	OR (95% CI)	*p* Value
Level 2 or worse	Level 1	2206	48.9			
Level 3 or worse	Level 1	2206	22.8			<0.001
	Level 2	4055	37.8	Level 1	2.0 (1.8, 2.3)	
Level 4 or worse	Level 1	2206	14.3			<0.001
	Level 2	4055	22.5	Level 1	1.7 (1.5, 1.9)	
	Level 3	2515	35.5	Level 2	1.9 (1.7, 2.1)	
				Per level	1.8 (1.7, 1.9)	<0.001
Level 5 or worse	Level 1	2206	7.9			<0.001
	Level 2	4055	11.3	Level 1	1.4 (1.2, 1.7)	
	Level 3	2515	18.5	Level 2	1.7 (1.5, 2.0)	
	Level 4	2370	24.5	Level 3	1.4 (1.2, 1.6)	
				Per level	1.5 (1.5, 1.6)	<0.001
Level 6 or worse	Level 1	2206	6.8			<0.001
	Level 2	4055	7.7	Level 1	1.2 (1.0, 1.4)	
	Level 3	2515	9.8	Level 2	1.3 (1.1, 1.5)	
	Level 4	2370	11.6	Level 3	1.2 (1.0, 1.4)	
	Level 5	2210	17.3	Level 4	1.5 (1.3, 1.8)	
				Per level	1.3 (1.2, 1.4)	<0.001
Any AMD	Level 1	2206	3.1			<0.001
	Level 2	4055	2.5	Level 1	0.8 (0.6, 1.1)	
	Level 3	2515	3.1	Level 2	1.2 (0.9, 1.6)	
	Level 4	2370	2.9	Level 3	0.9 (0.7, 1.3)	
	Level 5	2210	5.1	Level 4	1.8 (1.3, 2.4)	
	Level 6	2465	25.2	Level 5	5.5 (4.5, 6.8)	
				Per level	1.8 (1.7, 1.9)	<0.001

For ARMS2 A69S genotype (Figure 4), most of the strongest associations were for smaller areas of drusen at the start of an interval. Having two risk alleles was more strongly associated with incidence than having one risk allele present. For example, incidence of Level 6 was higher when there were two risk alleles compared to one or 0, but was only statistically significant when the initial level of drusen severity was Level 1, 2, or 3. Among individuals with Level 1, 13% of those with the T/T genotype (2 risk alleles), 8% of the G/T (1 risk allele) and 6% of those with G/G (0 risk alleles) developed Level 6 over the next five years (OR = 1.7 per risk allele, *p* = 0.002 after adjusting for age and sex). Among persons with Level 5, 17%, 17% and 23% of those with 0, 1 and 2 risk alleles, respectively, developed Level 6 (OR = 1.2 per risk allele, *p* = 0.16 after adjusting for age and sex).

## 4. Discussion

There is a general consensus that large soft drusen and pigmentary abnormalities define an early AMD phenotype and that neovascular AMD and pure geographic atrophy define the late advanced AMD phenotype. However, there is less agreement regarding whether a large area of small hard distinct drusen defines an even earlier AMD phenotype. In the Beaver Dam Eye Study, we found that the incidence of increasingly larger areas of small hard drusen (more severe level) is dependent upon the prior less severe level (smaller area) of small hard drusen. Even an eye with a minimal area of small hard drusen (Level 2) has approximately 50% greater odds of progressing by one step than an eye with no drusen (Level 1). While there is some variability, a stepwise increase in severity seems reasonable to assume. Furthermore, the data are consistent with the notion that there is an increasing likelihood of progression to intermediate drusen or any AMD with a larger area of small hard drusen. We had previously reported that a large area >9086 µm²) of small hard distinct drusen compared to a small area (≤2596 µm²) of involvement of these drusen in the absence of any other signs of AMD was associated with increased 15-year cumulative incidence of early AMD (18% *vs.* 5%) and late AMD (1.5% *vs.* 0.4%) [7]. This was consistent with earlier observations from clinical studies suggesting that eyes with large numbers of small hard drusen were at greater risk of developing AMD [29,30,31].

The process by which large soft drusen develop and their relation to small hard drusen is poorly understood [29,31,32,33]. Large soft drusen have been thought to result either from the aggregation of small hard drusen or in areas where small hard drusen appear to be absent. Hard and soft drusen have differing molecular components. Sarks *et al.* and others [14,34,35] have described the focal aggregation of small hard drusen into mounds of membranous debris, which accumulate as a widespread shallow layer between the basement membrane of the pigment epithelium and Bruch membrane. Small subclinical sized drusen (<30 µm in diameter) not usually visible on ophthalmoscopy have been found to become confluent and form soft drusen [31]. Areas surrounding individual large soft drusen have been shown to contain several small merging drusen and thick basal laminar drusen. Further understanding of the biochemical, physiological, and anatomic changes that take place as large soft drusen evolve is needed.

We wanted to examine whether age and different high risk AMD candidate genes might affect different stages of drusen development in addition to the natural history of drusen development. In the Beaver Dam Eye Study, genetic associations from CFH Y402H and ARMS2 A69S appear to affect different stages of drusen development. Some of these differences are related to underpowered tests with ARMS2 but may also represent differences in the genetic influences on disease.

In the present report, besides the differing associations of the SNPs of two genes associated with AMD (CFH Y402H rs1061190 and ARMS2 A69S rs10490924), we found relationships of age but not sex to the incidence of increasing areas of macular involvement with small hard drusen, intermediate drusen, and AMD. It is difficult to compare our findings with those of other population-based studies that have examined the incidence and progression of small hard distinct drusen due to differences in the criteria defining the drusen endpoint among the studies. In the Inter99 Eye Study [36], there was no relationship between the presence of 20 or more small hard macular drusen with gene SNPs CFH Y402H, LOC387715 A69S, HTRA1 rs11200638, CFB R32Q, and CFB L9H while drusen >63 µm in diameter were related to CFH Y402H. The Münster Aging and Retina Study (MARS) [37] examined drusen features such as number, confluence, type, size, and area occupied by macular drusen in 406 patients with early AMD and 170 healthy controls with a follow-up examination after 2.6 years. In that study, over a 2½ year period, CFH Y402H but not ARMS2 A69S was associated with the development of 20 or more small hard drusen in eyes described as healthy at baseline. In another study, investigators developing a new clinical classification system for AMD concluded that small drusen (<63 µm) should be considered to be a result of normal aging changes and that there was no clinically relevant increased risk of late AMD developing in these eyes [30]. In that study, persons with intermediate drusen (63 to 124 µm in diameter) were considered to have early AMD. These findings from the Beaver Dam Eye Study and other studies suggest that intermediate drusen may be one of the earliest stages of AMD clinically identifiable by grading of fundus photographs. Others have shown an increased risk of developing AMD when intermediate drusen are present [30,36,37].

Our study has many strengths, including repeated examinations during a 20-year period that used detailed standardized procedures for obtaining stereoscopic color fundus photographs of the macula and an objective system for grading those photographs for AMD phenotypes. However, the study also had a number of limitations. First, the Beaver Dam Eye Study cohort is racially/ethnically homogeneous (over 99% white), which limits our inferences regarding these findings in nonwhites. Second, we used SNPs from only two AMD candidate genes; it is possible that inclusion of all the identified possible loci may have affected our findings. Non-AMD related processes may cause the development of the first few small hard macular drusen. There may also be more misclassification in eyes with no or only small drusen because it is more difficult to detect some small hard drusen, leading to greater variability in grading their presence than in eyes with larger drusen or more severe late signs of AMD and resulting in detection bias due to size. Mortality may also have limited the interpretation of associations because of selective survival.

## 5. Conclusions

This report provides long-term population-based observations regarding the relationships of age, sex, and presence of risk alleles for two AMD candidate genes, CFH Y402H rs1061170 and ARMS2 A69S rs10490924, to the incidence of small hard drusen, as well as their role in the natural history of AMD in its earliest clinical stages using grading of stereoscopic 30° color fundus film photography. The findings from the Beaver Dam Eye Study and others provide some evidence that intermediate drusen and large areas of small hard drusen may be early clinically detectable stages in the trajectory of the disease and not just risk indicators. Intervals of observation of the cohort longer than five years may be needed to provide enough time to evaluate the natural history of no or a few small drusen progressing to AMD. This will be evaluated by multistate modeling in future analyses. Our findings also suggest the genetic risks from CFH and ARMS2 may influence different stages of the disease in different ways. Inclusion of newer technological assessment tools such as spectral domain optical coherence tomography and autofluorescence may provide more sensitive and reliable approaches to detect and quantitate even earlier stages of AMD [33]. This may prove useful in the study of pathogenetic risk factors only associated with these earlier stages of AMD. However, risks and benefits of defining an individual as having early AMD when newer classifications are used in clinical practice will need to be examined further.

## Supplementary Files

Supplementary File 1
