# Supplementary material for: Small Drusen and Age-Related Macular Degeneration: The Beaver Dam Eye Study
Source: J Clin Med. 2015 Mar 9;4(3):425–40. doi: 10.3390/jcm4030425 (PMC4402933; doi:10.3390/jcm4030425)
Supplement: Supplementary File 1 [file jcm-04-00425-s001.pdf]

Supplementary Information

Table S1. Five-year incidence of a minimal area of small hard drusen (Level 2 or worse) by drusen severity level at beginning of interval.

Risk Factor	Risk Level	*N* at Risk	% Incident	Comparison Level	OR (95% CI)	*p* value
Initial State: Level 1						
Age group, years	<60	772	47.2			0.05
	60–69	803	47.2	<60	1.0 (0.8, 1.2)	
	≥70	631	53.2	60–69	1.3 (1.0, 1.6)	
				Per group	1.1 (1.0, 1.3)	0.03
Sex	Female	1160	50.1			0.32
	Male	1046	47.6	Female	0.9 (0.8, 1.1)	
CFH genotype	T/T	850	48.2			0.23
	T/C	966	48.7	T/T	1.0 (0.8, 1.2)	
	C/C	225	54.2	T/C	1.3 (0.9, 1.7)	
				Per allele	1.1 (1.0, 1.3)	0.18
ARMS2 genotype	G/G	1280	48.1			0.17
	G/T	731	51.6	G/G	1.2 (1.0, 1.4)	
	T/T	69	42.0	G/T	0.7 (0.4, 1.2)	
				Per allele	1.1 (0.9, 1.3)	0.41

AMD, age-related macular degeneration; *ARMS2*, age-related maculopathy susceptibility 2 rs10490924; *CFH*, complement factor H rs1061170; CI, confidence interval; OR, odds ratio.

Table S2. Five-year incidence of a small area of small hard drusen (Level 3 or worse) by drusen severity level at beginning of interval.

Risk Factor	Risk Level	*N* at Risk	% Incident	Comparison Level	OR (95% CI)	*p* value
Initial State: Level 1						
Age group, years	<60	772	19.0			<0.001
	60–69	803	20.9	<60	1.1 (0.9, 1.4)	
	≥70	631	29.8	60–69	1.6 (1.3, 2.1)	
				Per group	1.3 (1.2, 1.5)	<0.001
Sex	Female	1160	25.2			0.02
	Male	1046	20.2	Female	0.8 (0.6, 1.0)	
CFH genotype	T/T	850	21.4			0.54
	T/C	966	23.3	T/T	1.1 (0.9, 1.4)	
	C/C	225	23.1	T/C	1.0 (0.7, 1.4)	
				Per allele	1.1 (0.9, 1.3)	0.32
ARMS2 genotype	G/G	1280	21.0			0.02
	G/T	731	26.4	G/G	1.4 (1.1, 1.7)	
	T/T	69	24.6	G/T	1.0 (0.5, 1.9)	
				Per allele	1.3 (1.1, 1.5)	0.008
Initial State: Level 2						
Age group, years	<60	1724	39.0			0.01
	60–69	1498	34.8	<60	0.8 (0.7, 1.0)	
	≥70	833	40.6	60–69	1.3 (1.1, 1.5)	

Table S2. *Cont.*

				Per group	1.0 (0.9, 1.1)	0.93
Sex	Female	2279	38.6			0.28
	Male	1776	36.8	Female	0.9 (0.8, 1.1)	
CFH genotype	T/T	1546	35.4			0.05
	T/C	1715	38.8	T/T	1.2 (1.0, 1.3)	
	C/C	512	40.6	T/C	1.1 (0.9, 1.3)	
				Per allele	1.1 (1.0, 1.2)	0.02
ARMS2 genotype	G/G	2360	35.6			<0.001
	G/T	1367	40.3	G/G	1.2 (1.1, 1.4)	
	T/T	141	48.9	G/T	1.4 (1.0, 2.0)	
				Per allele	1.3 (1.1, 1.4)	<0.001

AMD, age-related macular degeneration; *ARMS2*, age-related maculopathy susceptibility 2 rs10490924; *CFH*, complement factor H rs1061170; CI, confidence interval; OR, odds ratio.

Table S3. Five-year incidence of a moderate area of small hard drusen (Level 4 or worse) by drusen severity level at beginning of interval.

Risk Factor	Risk Level	*N* at Risk	% Incident	Comparison Level	OR (95% CI)	*p* value
Initial State: Level 1						
Age group, years	<60	772	9.5			<0.001
	60–69	803	12.8	<60	1.4 (1.0, 1.9)	
	≥70	631	22.2	60–69	2.0 (1.5, 2.6)	
				Per group	1.7 (1.4, 2.0)	<0.001
Sex	Female	1160	16.3			0.02
	Male	1046	12.1	Female	0.7 (0.6, 1.0)	
CFH genotype	T/T	850	12.5			0.15
	T/C	966	15.5	T/T	1.3 (1.0, 1.7)	
	C/C	225	13.3	T/C	0.9 (0.6, 1.4)	
				Per allele	1.1 (0.9, 1.4)	0.16
ARMS2 genotype	G/G	1280	12.3			0.008
	G/T	731	17.0	G/G	1.5 (1.1, 1.9)	
	T/T	69	18.8	G/T	1.4 (0.7, 2.7)	
				Per allele	1.4 (1.2, 1.8)	0.001
Initial State: Level 2						
Age group, years	<60	1724	19.4			<0.001
	60–69	1498	21.9	<60	1.2 (1.0, 1.4)	
	≥70	833	30.1	60–69	1.5 (1.3, 1.9)	
				Per group	1.3 (1.2, 1.5)	<0.001
Sex	Female	2279	23.7			0.07
	Male	1776	20.9	Female	0.9 (0.7, 1.0)	
CFH genotype	T/T	1546	22.2			0.58
	T/C	1715	22.1	T/T	1.0 (0.8, 1.2)	
	C/C	512	23.4	T/C	1.1 (0.9, 1.4)	
				Per allele	1.0 (0.9, 1.2)	0.43
ARMS2 genotype	G/G	2360	20.7			<0.001

Table S3. *Cont.*

	G/T	1367	24.4	G/G	1.2 (1.1, 1.5)	
	T/T	141	32.6	G/T	1.6 (1.1, 2.3)	
				Per allele	1.3 (1.1, 1.5)	<0.001
Initial State: Level 3						
Age group, years	<60	1142	33.2			0.01
	60–69	948	35.3	<60	1.1 (0.9, 1.3)	
	≥70	425	42.1	60–69	1.3 (1.0, 1.7)	
				Per group	1.2 (1.1, 1.3)	0.004
Sex	Female	1424	37.3			0.04
	Male	1091	33.2	Female	0.8 (0.7, 1.0)	
CFH genotype	T/T	901	35.4			0.88
	T/C	1123	35.4	T/T	1.0 (0.8, 1.2)	
	C/C	297	36.4	T/C	1.1 (0.8, 1.4)	
				Per allele	1.0 (0.9, 1.2)	0.70
ARMS2 genotype	G/G	1491	34.0			0.10
	G/T	828	37.4	G/G	1.2 (1.0, 1.4)	
	T/T	78	43.6	G/T	1.3 (0.8, 2.2)	
				Per allele	1.2 (1.0, 1.4)	0.03

AMD, age-related macular degeneration; *ARMS2*, age-related maculopathy susceptibility 2 rs10490924; *CFH*, complement factor H rs1061170; CI, confidence interval; OR, odds ratio.

Table S4. Five-year incidence of a large area of small hard drusen (Level 5 or worse) by drusen severity level at beginning of interval.

Risk Factor	Risk Level	*N* at Risk	% Incident	Comparison Level	OR (95% CI)	*p* value
Initial State: Level 1						
Age group, years	<60	772	3.6			<0.001
	60–69	803	5.5	<60	1.5 (0.9, 2.5)	
	≥70	631	16.3	60–69	3.4 (2.3, 5.0)	
				Per group	2.5 (2.0, 3.1)	<0.001
Sex	Female	1160	8.7			0.45
	Male	1046	7.1	Female	0.9 (0.6, 1.2)	
CFH genotype	T/T	850	6.1			0.03
	T/C	966	9.2	T/T	1.6 (1.1, 2.3)	
	C/C	225	8.9	T/C	1.1 (0.6, 1.8)	
				Per allele	1.4 (1.1, 1.7)	0.01
ARMS2 genotype	G/G	1280	6.6			0.007
	G/T	731	9.6	G/G	1.6 (1.1, 2.2)	
	T/T	69	14.5	G/T	2.2 (1.0, 4.7)	
				Per allele	1.7 (1.3, 2.3)	<0.001
Initial State: Level 2						
Age group, years	<60	1724	6.9			<0.001
	60–69	1498	12.0	<60	1.8 (1.4, 2.3)	
	≥70	833	19.0	60–69	1.7 (1.4, 2.2)	
				Per group	1.8 (1.6, 2.0)	<0.001

Table S4. *Cont.*

Sex	Female	2279	12.4			0.03
	Male	1776	9.8	Female	0.8 (0.6, 1.0)	
CFH genotype	T/T	1546	10.4			0.14
	T/C	1715	11.5	T/T	1.1 (0.9, 1.4)	
	C/C	512	12.5	T/C	1.2 (0.9, 1.6)	
				Per allele	1.2 (1.0, 1.4)	0.05
ARMS2 genotype	G/G	2360	10.0			<0.001
	G/T	1367	12.3	G/G	1.3 (1.0, 1.6)	
	T/T	141	21.3	G/T	2.2 (1.4, 3.4)	
				Per allele	1.4 (1.2, 1.7)	<0.001
Initial State: Level 3						
Age group, years	<60	1142	14.9			<0.001
	60–69	948	17.5	<60	1.2 (1.0, 1.5)	
	≥70	425	30.4	60–69	2.0 (1.5, 2.7)	
				Per group	1.5 (1.3, 1.8)	<0.001
Sex	Female	1424	19.7			0.12
	Male	1091	17.0	Female	0.8 (0.7, 1.0)	
CFH genotype	T/T	901	16.2			0.02
	T/C	1123	19.4	T/T	1.3 (1.0, 1.6)	
	C/C	297	21.9	T/C	1.2 (0.9, 1.7)	
				Per allele	1.3 (1.1, 1.5)	0.005
ARMS2 genotype	G/G	1491	17.0			0.06
	G/T	828	20.8	G/G	1.3 (1.0, 1.6)	
	T/T	78	24.4	G/T	1.3 (0.7, 2.3)	
				Per allele	1.3 (1.1, 1.5)	0.01
Initial State: Level 4						
Age group, years	<60	1040	20.7			<0.001
	60–69	886	24.6	<60	1.2 (1.0, 1.5)	
	≥70	444	33.3	60–69	1.5 (1.2, 2.0)	
				Per group	1.4 (1.2, 1.5)	<0.001
Sex	Female	1387	23.8			0.29
	Male	983	25.5	Female	1.1 (0.9, 1.3)	
CFH genotype	T/T	888	22.7			0.007
	T/C	1018	23.8	T/T	1.1 (0.9, 1.3)	
	C/C	268	31.7	T/C	1.6 (1.2, 2.1)	
				Per allele	1.2 (1.1, 1.4)	0.005
ARMS2 genotype	G/G	1362	25.0			0.13
	G/T	839	22.6	G/G	0.9 (0.7, 1.1)	
	T/T	69	33.3	G/T	1.7 (1.0, 3.0)	
				Per allele	1.0 (0.8, 1.2)	0.88

AMD, age-related macular degeneration; *ARMS2*, age-related maculopathy susceptibility 2 rs10490924; *CFH*, complement factor H rs1061170; CI, confidence interval; OR, odds ratio.

Table S5. Five-year incidence of intermediate drusen (Level 6 or worse) by drusen severity level at beginning of interval.

Risk Factor	Risk Level	*N* at Risk	% Incident	Comparison Level	OR (95% CI)	*p* value
Initial State: Level 1						
Age group, years	<60	772	2.8			<0.001
	60–69	803	4.5	<60	1.6 (0.9, 2.8)	
	≥70	631	14.4	60–69	3.6 (2.4, 5.4)	
				Per group	2.6 (2.0, 3.4)	<0.001
Sex	Female	1160	7.5			0.42
	Male	1046	5.9	Female	0.9 (0.6, 1.2)	
CFH genotype	T/T	850	5.6			0.20
	T/C	966	7.7	T/T	1.4 (1.0, 2.1)	
	C/C	225	6.7	T/C	0.9 (0.5, 1.7)	
				Per allele	1.2 (0.9, 1.6)	0.13
ARMS2 genotype	G/G	1280	5.7			0.02
	G/T	731	7.9	G/G	1.5 (1.0, 2.2)	
	T/T	69	13.0	G/T	2.4 (1.1, 5.4)	
				Per allele	1.7 (1.2, 2.3)	0.002
Initial State: Level 2						
Age group, years	<60	1724	4.5			<0.001
	60–69	1498	8.0	<60	1.8 (1.4, 2.5)	
	≥70	833	14.0	60–69	1.9 (1.4, 2.5)	
				Per group	1.9 (1.6, 2.2)	<0.001
Sex	Female	2279	8.3			0.24
	Male	1776	7.0	Female	0.9 (0.7, 1.1)	
CFH genotype	T/T	1546	7.0			0.24
	T/C	1715	7.9	T/T	1.2 (0.9, 1.5)	
	C/C	512	8.4	T/C	1.2 (0.8, 1.7)	
				Per allele	1.2 (1.0, 1.4)	0.09
ARMS2 genotype	G/G	2360	6.9			0.004
	G/T	1367	8.3	G/G	1.2 (0.9, 1.6)	
	T/T	141	16.3	G/T	2.5 (1.5, 4.1)	
				Per allele	1.4 (1.2, 1.8)	<0.001
Initial State: Level 3						
Age group, years	<60	1142	6.0			<0.001
	60–69	948	10.1	<60	1.8 (1.3, 2.5)	
	≥70	425	19.5	60–69	2.1 (1.5, 2.9)	
				Per group	1.9 (1.6, 2.3)	<0.001
Sex	Female	1424	9.8			0.78
	Male	1091	9.8	Female	1.0 (0.8, 1.4)	
CFH genotype	T/T	901	9.2			0.35
	T/C	1123	10.7	T/T	1.2 (0.9, 1.7)	
	C/C	297	9.4	T/C	0.9 (0.6, 1.5)	
				Per allele	1.1 (0.9, 1.4)	0.28
ARMS2 genotype	G/G	1491	8.3			0.01

Table S5. *Cont.*

	G/T	828	12.1	G/G	1.5 (1.1, 2.0)	
	T/T	78	16.7	G/T	1.6 (0.8, 3.2)	
				Per allele	1.5 (1.2, 1.9)	<0.001
Initial State: Level 4						
Age group, years	<60	1040	6.8			<0.001
	60–69	886	10.9	<60	1.7 (1.2, 2.3)	
	≥70	444	24.1	60–69	2.6 (1.9, 3.5)	
				Per group	2.1 (1.8, 2.5)	<0.001
Sex	Female	1387	11.9			0.57
	Male	983	11.2	Female	0.9 (0.7, 1.2)	
CFH genotype	T/T	888	10.0			<0.001
	T/C	1018	11.1	T/T	1.2 (0.9, 1.6)	
	C/C	268	18.7	T/C	2.1 (1.4, 3.1)	
				Per allele	1.5 (1.2, 1.8)	<0.001
ARMS2 genotype	G/G	1362	11.2			0.36
	G/T	839	11.4	G/G	1.0 (0.8, 1.3)	
	T/T	69	17.4	G/T	1.8 (0.9, 3.8)	
				Per allele	1.1 (0.9, 1.4)	0.36
Initial State: Level 5						
Age group, years	<60	1014	11.4			<0.001
	60–69	803	18.3	<60	1.7 (1.3, 2.3)	
	≥70	393	30.5	60–69	1.9 (1.5, 2.6)	
				Per group	1.8 (1.6, 2.1)	<0.001
Sex	Female	1281	18.3			0.37
	Male	929	15.9	Female	0.9 (0.7, 1.1)	
CFH genotype	T/T	833	14.3			<0.001
	T/C	937	17.0	T/T	1.2 (0.9, 1.6)	
	C/C	269	25.3	T/C	1.7 (1.2, 2.4)	
				Per allele	1.4 (1.2, 1.7)	<0.001
ARMS2 genotype	G/G	1294	16.8			0.23
	G/T	727	17.2	G/G	1.1 (0.8, 1.4)	
	T/T	92	22.8	G/T	1.6 (0.9, 2.7)	
				Per allele	1.2 (0.9, 1.4)	0.16

AMD, age-related macular degeneration; *ARMS2*, age-related maculopathy susceptibility 2 rs10490924; *CFH*, complement factor H rs1061170; CI, confidence interval; OR, odds ratio.

Table S6. Five-year incidence of any AMD by drusen severity level at beginning of interval.

Risk Factor	Risk Level	*N* at Risk	% Incident	Comparison Level	OR (95% CI)	*p* value
Initial State: Level 1						
Age group, years	<60	772	1.4			<0.001
	60–69	803	2.2	<60	1.6 (0.7, 3.5)	
	≥70	631	6.3	60–69	2.9 (1.6, 5.2)	
				Per group	2.3 (1.6, 3.3)	<0.001
Sex	Female	1160	3.4			0.84
	Male	1046	2.9	Female	1.0 (0.6, 1.5)	
CFH genotype	T/T	850	2.8			0.54
	T/C	966	3.7	T/T	1.3 (0.8, 2.3)	
	C/C	225	2.7	T/C	0.8 (0.3, 1.8)	
				Per allele	1.1 (0.8, 1.6)	0.58
ARMS2 genotype	G/G	1280	2.7			0.12
	G/T	731	3.7	G/G	1.4 (0.9, 2.4)	
	T/T	69	7.2	G/T	2.7 (1.0, 7.5)	
				Per allele	1.7 (1.1, 2.6)	0.02
Initial State: Level 2						
Age group, years	<60	1724	1.2			<0.001
	60–69	1498	2.6	<60	2.3 (1.3, 3.9)	
	≥70	833	5.2	60–69	2.0 (1.3, 3.2)	
				Per group	2.1 (1.6, 2.8)	<0.001
Sex	Female	2279	2.7			0.67
	Male	1776	2.3	Female	0.9 (0.6, 1.4)	
CFH genotype	T/T	1546	2.3			0.36
	T/C	1715	2.2	T/T	1.0 (0.6, 1.6)	
	C/C	512	3.1	T/C	1.6 (0.9, 2.9)	
				Per allele	1.2 (0.9, 1.7)	0.25
ARMS2 genotype	G/G	2360	2.2			0.11
	G/T	1367	2.9	G/G	1.3 (0.9, 2.1)	
	T/T	141	5.7	G/T	2.4 (1.0, 5.6)	
				Per allele	1.6 (1.1, 2.2)	0.02
Initial State: Level 3						
Age group, years	<60	1142	1.4			<0.001
	60–69	948	4.0	<60	3.2 (1.7, 5.9)	
	≥70	425	5.9	60–69	1.4 (0.9, 2.4)	
				Per group	2.1 (1.6, 2.7)	<0.001
Sex	Female	1424	3.2			0.87
	Male	1091	3.0	Female	1.0 (0.6, 1.5)	
CFH genotype	T/T	901	2.6			0.20
	T/C	1123	3.8	T/T	1.6 (0.9, 2.7)	
	C/C	297	2.7	T/C	0.7 (0.3, 1.6)	
				Per allele	1.2 (0.9, 1.7)	0.28
ARMS2 genotype	G/G	1491	2.7			0.44

Table S6. *Cont.*

	G/T	828	3.9	G/G	1.4 (0.8, 2.2)	
	T/T	78	3.8	G/T	1.1 (0.3, 3.6)	
				Per allele	1.3 (0.9, 1.9)	0.18
Initial State: Level 4						
Age group, years	<60	1040	1.3			<0.001
	60–69	886	2.4	<60	1.8 (0.9, 3.5)	
	≥70	444	7.7	60–69	3.4 (1.9, 6.0)	
				Per group	2.6 (1.8, 3.7)	<0.001
Sex	Female	1387	3.0			0.86
	Male	983	2.8	Female	1.0 (0.6, 1.6)	
CFH genotype	T/T	888	2.9			0.98
	T/C	1018	2.7	T/T	0.9 (0.5, 1.7)	
	C/C	268	2.6	T/C	1.1 (0.4, 2.5)	
				Per allele	1.0 (0.7, 1.5)	0.94
ARMS2 genotype	G/G	1362	2.9			0.71
	G/T	839	2.9	G/G	0.9 (0.6, 1.6)	
	T/T	69	1.4	G/T	0.5 (0.1, 4.3)	
				Per allele	0.9 (0.6, 1.4)	0.62
Initial State: Level 5						
Age group, years	<60	1014	2.0			<0.001
	60–69	803	5.0	<60	2.5 (1.5, 4.4)	
	≥70	393	13.2	60–69	2.9 (1.9, 4.5)	
				Per group	2.7 (2.1, 3.6)	<0.001
Sex	Female	1281	5.7			0.33
	Male	929	4.2	Female	0.8 (0.5, 1.2)	
CFH genotype	T/T	833	3.5			0.003
	T/C	937	5.3	T/T	1.5 (0.9, 2.5)	
	C/C	269	9.3	T/C	2.0 (1.2, 3.4)	
				Per allele	1.7 (1.3, 2.4)	<0.001
ARMS2 genotype	G/G	1294	5.1			0.75
	G/T	727	5.5	G/G	1.2 (0.8, 1.8)	
	T/T	92	5.4	G/T	1.1 (0.4, 2.9)	
				Per allele	1.1 (0.8, 1.6)	0.43
Initial State: Level 6						
Age group, years	<60	595	15.1			<0.001
	60–69	871	21.4	<60	1.6 (1.2, 2.1)	
	≥70	999	34.5	60–69	1.9 (1.6, 2.4)	
				Per group	1.8 (1.6, 2.0)	<0.001
Sex	Female	1485	25.6			0.71
	Male	980	24.6	Female	1.0 (0.9, 1.3)	
CFH genotype	T/T	924	20.2			<0.001
	T/C	1012	27.8	T/T	1.5 (1.2, 1.9)	
	C/C	348	30.5	T/C	1.3 (1.0, 1.7)	
				Per allele	1.4 (1.2, 1.6)	<0.001
ARMS2 genotype	G/G	1385	22.2			<0.001
	G/T	831	28.0	G/G	1.5 (1.2, 1.8)	

Table S6. *Cont.*

	T/T	125	42.4	G/T	1.9 (1.2, 2.8)	
				Per allele	1.6 (1.3, 1.9)	<0.001

AMD, age-related macular degeneration; *ARMS2*, age-related maculopathy susceptibility 2 rs10490924; *CFH*, complement factor H rs1061170; CI, confidence interval; OR, odds ratio.
